# Hand-grip test is a good predictor of extubation success in adult ICU patients

**DOI:** 10.1186/cc9581

**Published:** 2011-03-11

**Authors:** D De Bels, J Devriendt, P Gottignies, D Chochrad, S Theunissen, T Snoeck, C Balestra, U Pilard, S Roques

**Affiliations:** 1 Brugmann University Hospital, Brussels, Belgium; 2 Hôpitaux IRIS Sud, Brussels, Belgium; 3 ISEK Environmental Physiology Laboratory, Brussels, Belgium

## Introduction

Ventilator weaning protocols have been published during the past 20 years. Although patients fulfill weaning criteria, they may still experience extubation failure. Risk factors include respiratory muscle weakness. This is accompanied by peripheral muscle weakness. The aim of the study is to evaluate the possible relation between peripheral (hand) muscle strength and extubation success in ICU patients.

## Methods

Fifty-four consecutive patients (62 ± 14 years) extubated in the ICUs of the Brugmann University Hospital and the Etterbeek-Ixelles General Hospital were included in the study. Extubation failure was defined as reintubation within 48 hours after extubation. Hand muscle strength is measured by a grip test method.

## Results

**Figure 1:**
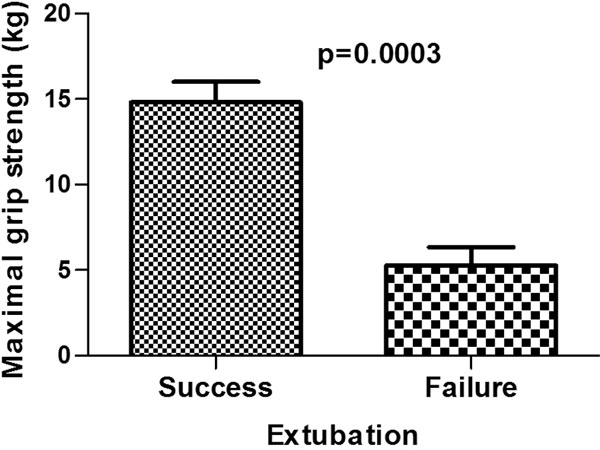
**Difference between maximal grip forces**.

Maximal hand grip strength is statistically (14.8 ± 7.7 vs. 5.3 ± 3.8 kg, *P *< 0.001) higher in patients successfully undergoing extubation compared with patients failing extubation. See Figure 1.

## Conclusions

Hand grip strength testing is a good predictor of successful extubation in ICU patients. The positive predictive value of 100% is obtained if maximal strength is >13 kg. Further studies are needed before grip testing could be routinely used as a decision-making test for extubation in ICU patients.

